# Supplementary material for: Inhibitory Effects of 2N1HIA (2-(3-(2-Fluoro-4-Methoxyphenyl)-6-Oxo-1(6H)-Pyridazinyl)-N-1H-Indol-5-Ylacetamide) on Osteoclast Differentiation via Suppressing Cathepsin K Expression
Source: Molecules. 2018 Nov 29;23(12):3139. doi: 10.3390/molecules23123139 (PMC6321589; doi:10.3390/molecules23123139)
Supplement: Supplementary file 1 [file molecules-23-03139-s001.pdf]

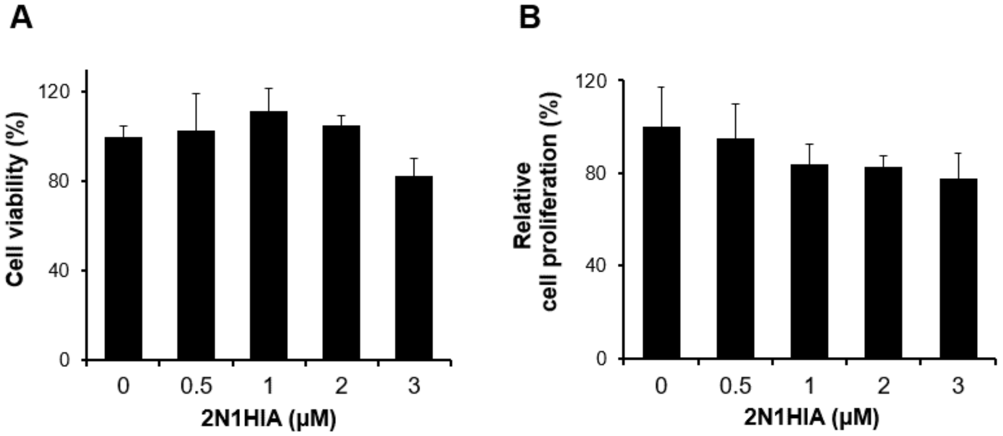

Figure S1. Cell viability and cell proliferation were measured using the MTT (A) and BrdU (B) assays, respectively. Data are presented as mean ± SD from three independent experiments; * $p < 0.05$, ** $p < 0.01$ (comparison to the control 100%).

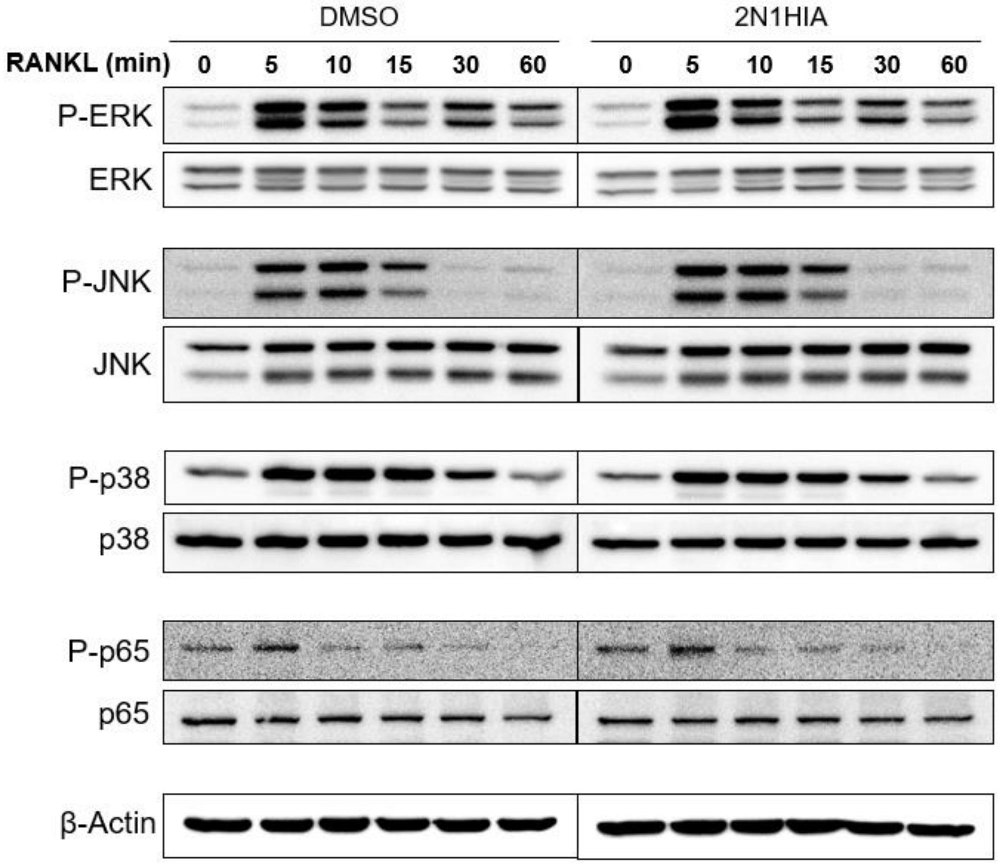

Figure S2. Effects of 2N1HIA on RANKL-induced signals in BMMs. M-CSF treated BMMs were pre-treated with 1 μM of 2N1HIA or control (DMSO) for 10 min and RANKL (50 ng/mL) was used to stimulate cells at the indicated times. Cells were analyzed via western blotting to detect anti-phospho-ERK, JNK, p38, and p65 signal proteins, as indicated.

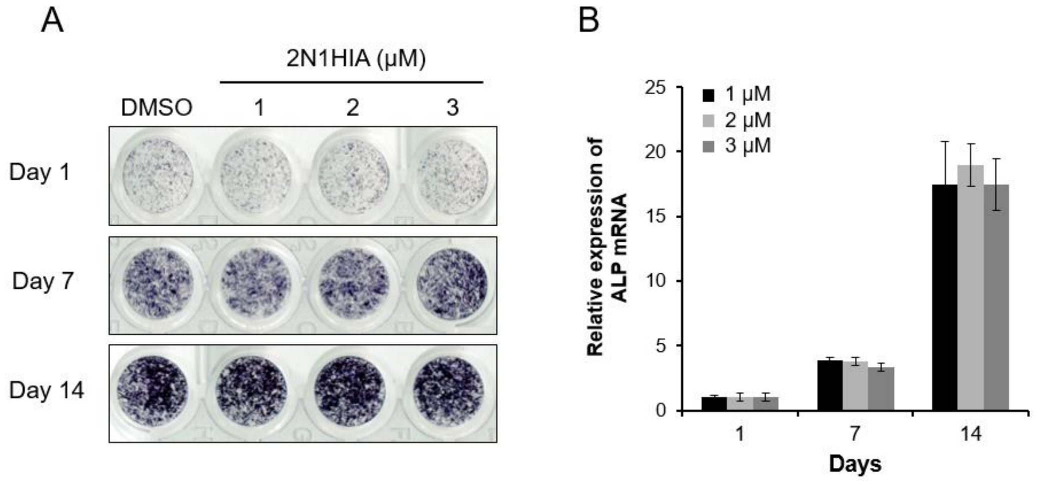

Figure S3. Effects of 2N1HIA on BMP-induced osteoblast differentiation. (A) Calvarial osteoblasts were cultured for different periods of time and then stained for ALP. (B) At the indicated time points, ALP mRNA was analyzed using real-time PCR. The expression was normalized to glyceraldehyde 3-phosphate dehydrogenase (GAPDH) and presented relative to day one.

Table S1. Compounds used in the study.

No.	Name	Molecular weight (kDa)	Catalog number
1	2-[3-(2-fluoro-4-methoxyphenyl)-6-oxo-1(6H)-pyridazinyl]-N1H-indol-5-ylacetamide	392.38	9309204
2	2-[3-(2-fluorophenyl)-6-oxo-1(6H)-pyridazinyl]-N-phenylacetamide	323.32	9288567
3	2-[3-(3-methoxyphenyl)-6-oxo-1(6H)-pyridazinyl]-N-phenylacetamide	335.36	9329589
4	N-(2-chlorophenyl)-2-(6-oxo-3-phenyl-1(6H)-pyridazinyl)acetamide	339.78	9328644
5	N-(2,4-difluorophenyl)-2-(6-oxo-3-phenyl-1(6H)-pyridazinyl)acetamide	341.31	9217398
6	N-(2-fluorophenyl)-2-[3-(2-fluorophenyl)-6-oxo-1(6H)-pyridazinyl]acetamide	341.31	9338014
7	N-1H-indol-5-yl-2-(6-oxo-3-phenyl-1(6H)-pyridazinyl)acetamide	344.37	9314216
8	N-1H-indol-6-yl-2-(6-oxo-3-phenyl-1(6H)-	344.37	9324040

	pyridazinyl)acetamide		
9	2-[2-(2,3-dihydro-1H-indol-1-yl)-1-methyl-2-oxoethyl]-6-phenyl-3(2H)-pyridazinone	345.39	9192738
10	4-{[(6-oxo-3-phenyl-1(6H)-pyridazinyl)acetyl]amino}benzamide	348.36	9317292
11	N-1,3-benzodioxol-5-yl-2-(6-oxo-3-phenyl-1(6H)-pyridazinyl)acetamide	349.34	9233542

Table S2. Primers used in the study.

Gene	Primer sequence (5′→3′)	
Cathepsin K	Forward	GGACGCAGCGATGCTAACTAA
	Reverse	CAGAGAGAAGGGAAGTAGAGTTGTCACT
CD47	Forward	TTTGGTCGGGCTGTGTCTCT
	Reverse	GCTATGATCCCCAAACCTGAAA
DC-STAMP	Forward	CGCACGATGCTTCATTCTTC
	Reverse	CAGTGCCAGCCGCAATC
GAPDH	Forward	TGTGTCCGTCGTGGATCTGA
	Reverse	GATGCCTGCTTCACCACCTT
MMP9	Forward	CTGGACAGCCAGACACTAAAG
	Reverse	CTCGCGGCAAGTCTTCAGAG
NFATc1	Forward	ACCACCTTTCCGCAACCA
	Reverse	GGTACTGGCTTCTCTTCCGTTTC
OC-STAMP	Forward	ACTATGGCCACCCGGGAAT
	Reverse	GGCCCAAGGGAGTCATGTG
OSCAR	Forward	TGGCGGTTTGCACTCTTCA
	Reverse	GGAAGAACTCAGCCAGCTCAA
RANK	Forward	CGACTGGTTCACTGCTCCTAATC
	Reverse	CTGTCGTTCTCCCCCACTTC
TRAF6	Forward	TCGGACCCTGGAGGACAA
	Reverse	CCAAACTTGCCAATCTTCCAA
ALP	Forward	AGTTCAGTGCGGTTCCAGACA
	Reverse	TGGCCTGGATCTCATCAGTATTT
